# Obstetric-Focused POCUS Training for Medical Students

**DOI:** 10.24908/pocus.v8i2.16316

**Published:** 2023-11-27

**Authors:** Koral Cohen, Jennifer Kidd, Emily Schiller, Agata Kantorowska, Wendy Kinzler, Martin Chavez

**Affiliations:** 1 NYU Grossman School of Medicine Mineola, NY USA; 2 Department of Obstetrics and Gynecology, NYU Langone Health-Long Island Mineola, NY USA; 3 Northwell Health, Donald and Barbara Zucker School of Medicine at Hofstra/Northwell Hempstead, NY USA

**Keywords:** Innovative Curricula, Technology in Education, UME / GME / CME Continuum

## Abstract

Point of care ultrasound (POCUS) is rapidly expanding throughout the United States. Due to its ability to quickly and accurately diagnose and guide therapy for critical conditions, POCUS is becoming routine in many specialties, with established guidelines in fields such as emergency medicine and critical care 1, 2, 3. For example, a study entitled “Ultrasound Integration in Undergraduate Medical Education: Comparison of Ultrasound Proficiency Between Trained and Untrained Medical Students” initiated an Emergency Medicine POCUS curriculum for first-year medical students that showed an increase in ultrasound capability 4. In short, as POCUS becomes more common practice, medical schools are beginning to implement POCUS training into their undergraduate medical education; studies from these institutions demonstrate that implementing a formal ultrasound curriculum into preclinical medical education significantly increases medical students’ POCUS capabilities^4^, ^5^ and assisted in their understanding and learning of anatomy 6, 7.

## Introduction

To the authors’ knowledge, there has not been a study that focused on integrating Obstetrics and Gynecology (OBGYN) POCUS curriculum within the medical school’s curriculum. An OBGYN Ultrasound Lecture Series was created for Ob/Gyn residents and was designed by the American Institute of Ultrasound Medicine (AIUM) 8. The 32 videos offered by this Lecture Series cover topics within OBGYN, Obstetric Imaging, and Gynecologic Imaging 8. These educational, in-depth videos may not be easily understandable/digestible for beginners in this field. They are very detailed and provide an extensive level of knowledge for residents. However, this results in a gap between medical students with limited knowledge and curricula with an extensive knowledge base. There are no widely available curricula to serve as an introduction for medical students of obstetrics imaging to assist in the closure of this educational gap. 

We describe a beginner-level, student-led para-curricular POCUS obstetric imaging workshop on obstetric imaging led by medical students. This workshop and curriculum were made assuming that students have no prior experience with obstetric imaging and, in general, have limited experience with ultrasound. In addition, the assumption was also made that these medical students have had limited clinical experience. Hence, this workshop should be educationally fit for any medical student level and aimed at beginners in obstetric imaging. This workshop also acknowledges the increasing importance of POCUS proficiency in medical professionals. Still, many medical schools struggle to find time in their curriculum and trained faculty to implement POCUS training programs 9. At our institution, students have limited exposure to POCUS throughout a 3-year, accelerated curriculum. Students are eager to gain skills in POCUS, which led to a student-led initiative. 

## Curricula

At our institution, medical students complete a 3-year accelerated curriculum. During year 1, the preclinical year, students attend radiology lectures that complement an organ-system-based curriculum twice weekly. During these radiology lectures, students mainly review various imaging modalities, including x-ray, CT, and ultrasound of adults, to understand the anatomy and pathology of multiple organs such as gallbladder, thyroid, testicles, and vasculature. First-year students do not learn how to utilize ultrasound probes or POCUS devices. POCUS is available for second-year students. It is introduced to second-year medical students participating in clinical rotations (MS2 students) through a one-day POCUS workshop focusing on venous compression ultrasound, lung assessment, and the Focused Assessment with Sonography in Trauma (FAST) exam. There is no formal POCUS training on obstetric ultrasound in the three-year curriculum. In addition, there is no formal POCUS training integrated into any clinical rotations. However, throughout medical school's second and third years, students may be exposed to POCUS in various settings throughout their clinical rotations. For example, during the 6-week Obstetrics and Gynecology rotation, Phase 2 medical students utilize ultrasound frequently for fetal assessment to detect fetal head. Still, there have been no formal curricula that cover how to do so. 

At our three-year accelerated medical school, medical students interested in pursuing OBGYN residency training recognized the benefit of early training in POCUS. They organized a series of POCUS workshops utilizing a handheld ultrasound device. Medical students interested in applying to an OBGYN residency met at the Maternal Fetal Medicine (MFM) sonography unit. This workshop was outside scheduled didactic teaching, and clinical rotation responsibilities and participation were voluntary. Before the first workshop, students were provided with an obstetric POCUS primer. This short video lecture outlined ultrasound science and the advantages of POCUS technology. The lecture concluded with the specific knowledge addressed during the obstetric ultrasound workshop: detect a fetal heart rate, confirm fetal head location, identify placental location, and identify a maximum vertical pocket of amniotic fluid. In addition, before the workshop, students completed a workshop gauging their interests in ultrasound training, career/specialty selection, prior experience with ultrasounds, and comfort and understanding of identifying specific structures (Table 1). 

**Table 1:** Survey administered prior to workshop

-	**1 = Disagree**	**2 = Neutral**	**3= Agree**
**I feel confident in my understanding of how ultrasound works.**	4	3	1
**I feel enthusiastic about the use of point-of-care ultrasound.**	0	2	6
**I feel comfortable detecting fetal heart rate.**	6	1	1
**I feel comfortable detecting fetal head location.**	6	1	1
**I feel comfortable detecting placenta location.**	6	1	1
I feel comfortable detecting maximum vertical pocket of amniotic fluid.	6	2	0
**I feel comfortable triaging labor and delivery patients.**	6	1	1
**I know how to apply proper probe techniques to a fetal ultrasound scan.**	6	1	1
**I feel comfortable performing a fetal ultrasound scan.**	6	2	0
**I intend to continue learning about ultrasound and refining my skills.**	0	0	8

**Figure 1:**
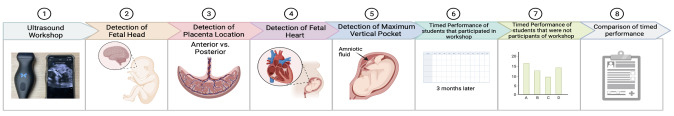
Summary of Methods.

The POCUS workshop was a 60-minute, hands-on, skills-based session led by maternal-fetal medicine faculty. Students learned four core skills that included how to 1) detect a fetal heart, 2) confirm fetal head location, 3) identify placental location, and 4) identify a maximum vertical pocket of amniotic fluid (Figure 1). Six medical students participated in this workshop. The faculty asked patients if they were interested in participating in medical student education. Patients had the teaching part of their ultrasound after the scheduled care portion was completed. Each student had approximately 6 minutes of personal hands-on scanning under the direct observation of the MFM faculty, allowing for real-time feedback and instruction. Three months later, the students that participated in the workshop, along with an additional six students that did not attend the workshop, performed fetal POCUS in the labor and delivery triage as a follow-up from the original workshop. Students were compared on their ability to perform all four core skills within five minutes. 

## Follow up

The initiative of implementing ultrasound workshops in medical schools through student-led interest groups was a success, with 100% attendance (8 students) from the OBGYN interest group reported. Six of the students were first-year medical students, and 2 of the students were second-year medical students. Six of the students were solely interested in pursuing OBGYN residencies, and 2 of the students were exploring OBGYN and one other field in medicine, either Internal Medicine or Surgery. Four of the students reported some prior experience with ultrasound, and 4 of the students reported no prior experience with ultrasounds. Two of the students had completed the OBGYN rotation (Phase 2), and all eight students had completed their Endocrine and Reproduction course (Phase 1). Students completed a survey before the workshop. Six out of the eight students agreed that they felt “enthusiastic about the use of POCUS,” and two reported neutral regarding the statement. 100% of students reported that they intend to continue learning about ultrasound and refining their skills. Six of the eight students said they felt uncomfortable detecting fetal heart rate, fetal head location, placental location, and maximum vertical pocket before the workshop (Table 1). 

**Figure 2:**
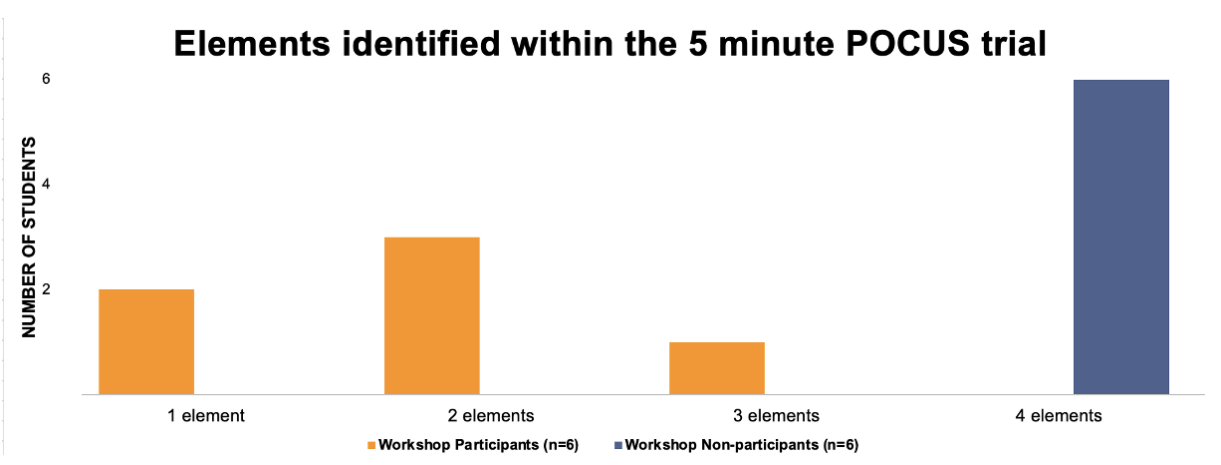
Summary of workshop results. Comparison of students, by participation status, in completing imaging elements identified during time constraint.

Six out of the eight students that participated in the workshop were assessed three months later. An MFM fellow timed the student while the student was tasked with identifying the four imaging skills they were taught in the workshop on patients in the Labor and Delivery triage. Patients consented to this before medical students utilized the POCUS device. The average time it took the students who completed the workshop to identify all four elements was 2 minutes and 36 seconds. The six students that served as controls were given five minutes to recognize the four imaging skills, and none could complete all four skills in the five minutes. Two out of 6 students identified one element, and 1 out of the six identified three elements (Figure 2).

## Discussion

The results of our study show that the creation of POCUS workshops is an accessible and effective way of retaining obstetric POCUS skills. Students that participated in the workshop were not only able to identify the four elements but were also able to efficiently identify them within a time constraint. In contrast, students who did not participate in the workshop could not locate all of the imaging elements within the time constraint. Initiating and implementing student-driven ultrasound workshops can be an effective way of providing valuable hands-on experiences to students. Additional POCUS workshops during the Endocrine and Reproduction course for first-year medical students in Phase 1 of the medical school’s curriculum and during the orientation of OBGYN rotation in Phase 2 will take place during the 2023-2024 academic year. These planned workshops will occur similarly to our original approach with additional feedback from students to continue expanding and improving this POCUS obstetric curriculum.

Student-initiated workshops introducing POCUS training early in medical school can serve as a helpful tool, particularly for students interested in OBGYN residencies. Future workshops will help further integrate POCUS education across preclinical and clinical years. Ultrasound exposure is integrated into our institution’s three-year curriculum. In the first year, students learn to interpret ultrasound images of the gallbladder, testicles, thyroid, and vasculature during an organ system-based preclinical year. However, this is from traditional ultrasound machines and not with the utility of POCUS. In year 2, students receive a 1-day POCUS training in venous compression ultrasound, lung assessment, and the FAST exam and may get exposure throughout the clinical rotations. Paracurricular, student-led obstetric-focused POCUS workshops could be integrated into a medical student curriculum to increase familiarity with POCUS in obstetric care. 

Integration of ultrasound training and POCUS training is vital in medical education to par with current technology and educate students to integrate this technology into clinical care. Integrating ultrasound training into student-led interest groups can be an excellent way for students and faculty to collaborate and create learning objectives aligned with students’ interests. In addition, ultrasound workshops can empower students to advance POCUS knowledge and utility across rotations. This early POCUS exposure in medical education could improve future training as these students transition to residents. Student-led initiatives such as this obstetric-focused POCUS workshop are supplementing medical student curricula. This could transition to a formalized portion of the medical student curricula to address potential gaps in ultrasound knowledge for a more graduated learning process. This knowledge provides priming for resident-level education and competency. By participating in these workshops, medical students increase their obstetric skills for residency and familiarity with the obstetric use of POCUS.

## Disclosures

Dr. Chavez has engaged in consulting work for Samsung Corporation. The authors report no additional disclosures relevant to this article. 
